# The association between cognitive impairment and breast and colorectal cancer screening utilization

**DOI:** 10.1186/s12885-021-08321-6

**Published:** 2021-05-12

**Authors:** Shuang Yang, Jiang Bian, Thomas J. George, Karen Daily, Dongyu Zhang, Dejana Braithwaite, Yi Guo

**Affiliations:** 1 Department of Health Outcomes and Biomedical Informatics, College of Medicine, University of Florida, 2004 Mowry Road, PO Box 100177, Gainesville, FL 32610 USA; 2 Cancer Informatics Shared Resource, University of Florida Health Cancer Center, Gainesville, FL USA; 3 Department of Medicine, Division of Hematology & Oncology, College of Medicine, University of Florida, Gainesville, FL USA; 4 Department of Epidemiology, College of Public Health and Health Professions, University of Florida, Gainesville, FL USA; 5 Department of Aging and Geriatric Research, College of Medicine, University of Florida, Gainesville, FL USA

**Keywords:** Dementia, Alzheimer’s disease, Mammogram, Colonoscopy, Disparity

## Abstract

**Background:**

Undergoing cancer screening is a debatable topic in patients with cognitive impairment. In this study, we aimed to examine the utilization and predictors of breast and colorectal cancer screening among screening eligible, cognitively impaired individuals.

**Methods:**

We analyzed the 2018 and 2019 National Health Interview Survey data (*n* = 12,965 and 24,782, respectively) on individuals eligible for breast or colorectal cancer screening. We calculated the percentage of cancer screening eligible individuals who received mammogram or colonoscopy by cognitive impairment status. We used multivariable logistic regression to examine whether having a recent mammogram or colonoscopy differed by cognitive impairment status, adjusting for covariates.

**Results:**

We observed a significantly lower percentage of mammogram use in the screening eligible, cognitively impaired (mild or severe) versus unimpaired women. Adjusting for the covariates, the cognitively impaired women, mild (odds ratio [OR] = 0.85; *p* = 0.015) or severe (OR = 0.54; *p* <  0.001), were less likely to have had a recent mammogram compared to the cognitively unimpaired women. Although statistically non-significant, the percentage of colonoscopy use in the screening eligible, cognitively impaired individuals were slightly higher than that in the cognitively unimpaired individuals. In the regression analysis, we found the cognitively impaired men, mild (OR = 0.79; *p* <  0.001) or severe (OR = 0.69; *p* = 0.038), were less likely to have had a recent colonoscopy compared to the cognitively unimpaired men. More studies are needed to examine the multilevel factors that underpin the difference in cancer screening utilization in this vulnerable population.

**Conclusion:**

Our results highlight the need for additional research to address utilization and effectiveness of cancer screening in individuals with cognitive impairment.

## Introduction

Cancer is the second leading cause of death in the United States (US) [1]. It is estimated that there will be 1,806,590 new cancer cases and 606,520 cancer deaths in the US in 2020 [2]. To improve cancer survival, detecting cancer early through screening tests is crucial as it provides the best opportunity for successful treatment and prognosis. The benefit of cancer screening tests in relation to survival has been well documented in many randomized clinical trials and observational studies [3–6]. For example, a meta-analysis of randomized clinical trials that used an intention-to-treat analysis found biennial mammogram could reduce the lifetime risk of breast cancer mortality among women aged 50 to 69 years exposed to screening by 19 to 22% [6]. A large prospective cohort study found colonoscopy could lower the chance of distal and proximal colorectal cancer mortality by 15.3 and 32.0%, respectively [3]. Given the benefit of cancer screening, national professional associations such as the US Preventive Services Task Force (USPSTF) and American Cancer Society have developed guidelines to recommend cancer screening in at-risk populations for lung, breast, colorectal, cervical, and prostate cancers.

On the other hand, even with the evidence-based screening guidelines, a decision to undergo cancer screening is not always straightforward, especially for patients with cognitive impairment which include Alzheimer’s disease and related dementias (ADRD). cognitive impairment is one of the earliest noticeable symptoms of ADRD [7], it was defined as “having trouble remembering, learning new things, concentrating, or making decisions that affect their everyday life” [8]. The 2015–2018 Behavioral Risk Factor Surveillance System (BRFSS) data estimated the prevalence of subjective cognitive impairment is about 11% in Americans aged 45 or older [9]. Studies show that about one in three individuals with cognitive impairment will develop ADRD in 5 years [10]. Patients with cognitive impairment and ADRD suffer from poor functional status, comorbid illnesses, and shortened life expectancy [11, 12]. It is therefore unclear whether cancer screening is useful for these individuals or whether they should undergo cancer screening, if otherwise eligible. Some evidence suggests that cancer screening in cognitively impaired older adults has survival benefit [13, 14], while other studies recommend that the cancer screening should be avoided by these individuals due to reduced life expectancy and potential harm from cancer screening procedures [15, 16]. Due to the lack of evidence on the effectiveness of cancer screening in patients with cognitive impairment and ADRD, none of the existing cancer screening guidelines provide specific recommendations for these individuals.

As the US population rapidly ages, the number of Americans who live with cognitive impairment and are simultaneously eligible for cancer screening will continue to grow. According to US Census data, the number of Americans aged 50 and older exceeded 117 million in 2019. By 2050, this number is expected to reach 157 million and accounting for 35% of the total population, which means almost one in every three Americans will be 50 years and older [17]. Considering that undergoing cancer screening is a debatable topic in patients with cognitive impairment and ADRD, it is important to examine the current status of cancer screening in these patients. In this study, we aimed to examine the utilization pattern of breast and colorectal cancer screening by status of cognitive impairment among screening eligible individuals.

## Methods

### Data source and study population

We used the 2018 and 2019 National Health Interview Survey (NHIS) data for this study. The NHIS is an annual, nationwide cross-sectional in-person survey of the noninstitutionalized US population. It collects data on a broad range of health topics and is widely used in public health research to monitor trends of illnesses and disabilities in the US. Our study analyzed data from the NHIS Sample Adult module, which contains in-depth information on demographics, health status, health care services and health behaviors. In the NHIS Sample Adult module, one adult per family is randomly selected to be interviewed. In the 2018 and 2019 NHIS data, the Sample Adult component included 25,417 and 31,997 adults, respectively. The NHIS provided weights that consider selection probability and nonresponse for calculating nationally representative estimates. This study was based on publicly available anonymized databases, and thus exempt from ethical compliance.

Our study populations were NHIS respondents who were eligible for breast or colorectal cancer screening based on the USPSTF cancer screening guidelines [3, 6]. The USPSTF recommends mammogram for women aged 50–74 years every 2 years, and colonoscopy for adults aged 50–75 years every 10 years. The 2018 and 2019 NHIS examined the utilization of screening tests for breast, colorectal, cervical, and prostate cancers. We chose to exclude cervical and prostate cancers from our analysis because (1) cervical cancer screening targets a much younger population among whom cognitive impairment is uncommon, and (2) the USPSTF concludes that the prostate-specific antigen (PSA)-based screening for prostate cancer is only beneficial in certain men, and recommends against regular prostate cancer screening.

### Outcomes and exposure

Our outcomes were (1) having a recent mammogram among women eligible for breast cancer screening, and (2) having a recent colonoscopy among adults eligible for colorectal cancer screening. In the NHIS, the participants were asked “have you had the test?” for each of the cancer screening tests, with the responses being “Yes” or “No”. For respondents who reported having had the screening test, a second question was asked about the most recent test: “*When did you have your most recent …*” . We defined women with a recent mammogram as those who indicated having had a mammogram in the first question and if the test was within 2 years in the second question. We defined adults with a recent colonoscopy as those who indicated having had a colonoscopy in the first question and if the test was within 10 years in the second question.

Our exposure of interest was cognitive impairment. The respondents were asked “*Do you have difficulty remembering or concentrating*?” with the responses being “*No difficulty*”, “*Some difficulty*”, “*A lot of difficulty*”, or “*Cannot do at all/unable to do*”. We categorized the respondents into 3 groups: *unimpaired*, those who chose “*No difficulty*”; *mildly impaired*, those who chose “*Some difficulty*”; and *severely impaired*, those who chose “*A lot of difficulty*” or “*Cannot do at all/unable to do*”. Respondents who selected the last two response categories were combined due to small sample sizes.

### Covariates

We included a number of covariates potentially related to having a recent mammogram or colonoscopy and cognitive impairment in our multivariable analysis, including age (5-year age intervals), sex (men or women), race-ethnicity, marital status, education, insurance coverage, and disease history. Race-ethnicity was categorized into *Non-Hispanic White*, *Non-Hispanic Black*, *Non-Hispanic Other*, and *Hispanic*. Marital status was categorized into single, live with spouse, and any other status. Educational attainment was categorized into *high school or less* and *more than high school*. Health insurance coverage was categorized into *insured* and *uninsured*. Lastly, we created a chronic disease score by summing the number of chronic diseases reported by the respondents. The respondents were asked “*Have you EVER been told by a doctor or health professional that you have …*? ” for a number of chronic diseases including hypertension, high cholesterol, coronary heart disease, angina, myocardial infarction, stroke, chronic obstructive pulmonary disease, asthma, diabetes, and arthritis. The chronic disease score ranged from 0 to 10, with higher scores indicating more chronic diseases. We created a separate indicator variable of cancer diagnosis history because having a cancer diagnosis was reported to be strongly associated with one’s cancer screening behavior [18–20].

### Statistical analysis

First, we examined differences in the covariates by cognitive impairment status (impaired versus unimpaired) using one-way ANOVA test for continuous variables and chi-squared tests for categorical variables. Second, we calculated the percentage of respondents eligible for mammogram or colonoscopy who took a recent screening test. The percentage was calculated as the number of the respondents who met the USPSTF cancer screening criteria and received a recent cancer screening test divided by the number of all respondents who met the USPSTF cancer screening criteria. We compared the percentages of cancer screening use in the impaired versus unimpaired respondents using the chi-squared test. Lastly, we examined the association of cognitive impairment with receipt of a cancer screening test in multivariable analyses. We built weighted multivariable logistic regression models with receipt of a recent cancer screening test as the dependent variable and cognitive impairment as the independent variable, adjusting for age, sex (for colonoscopy only), race-ethnicity, marital status, education, insurance coverage, cancer history, and chronic disease score. Due to the well-documented sex differences in colorectal cancer screening, we included and tested sex-by-covariate interactions in the logistic model for colonoscopy [21–23]. We used SAS 9.4 (Cary, NC) for all data analysis. We performed all analyses with the SURVEY procedures and incorporated population-based sampling weights provided by the NHIS. We calculated two-sided *p*-values for all statistics and considered a significance level of 0.05.

## Results

### Respondents’ characteristics

We summarized the respondents’ characteristics stratified by cognitive impairment status in Table 1. Our study populations included 12,965 women eligible for screening mammogram and 24,782 individuals eligible for colonoscopy. Among the 12,965 women eligible for mammogram, 2704 (or 20.9%) were cognitively impaired, with 2407 (18.6%) and 297 (2.3%) having mild and severe cognitive impairment, respectively. Overall, 72.6% of the women were Non-Hispanic White, 65.3% had more than high school education, 51.9% were living with spouse, 94.5% had health insurance, and 17.0% had a prior cancer diagnosis. Except for race/ethnicity and insurance coverage, each of the characteristic significantly differed by cognitive impairment status. Compared to cognitively unimpaired women, women with mild or severe impairment were more likely to have high school or less education (40.9% or 47.5% in the mildly or severely impaired vs. 32.9% in the unimpaired; *p* <  0.001), be single (10.0% or 12.6% vs. 9.2%; *p* <  0.001), have a prior cancer diagnosis (23.0% or 20.5% vs. 15.5%; *p* <  0.001), and have higher number of chronic diseases (2.62 or 3.12 vs. 1.62; *p* <  0.001).

**Table 1:** Respondents’ characteristics

	Mammogram					Colonoscopy				
	Overall N = 12,965 (100%)	Unimpaired *N* = 10,261 (79.1%)	Mildly impaired *N* = 2407 (18.6%)	Severely impaired *N* = 297 (2.3%)	***p***-value^**f**^	Overall N = 24,782 (100%)	Unimpaired *N* = 19,914 (80.4%)	Mildly impaired *N* = 4338 (17.5%)	Severely impaired *N* = 530 (2.1%)	***p-***value^**f**^
**Age**										
50–54	2278 (17.6%)	1858 (18.1%)	352 (14.6%)	68 (22.9%)	< 0.001	4405 (17.8%)	3674 (18.5%)	628 (14.5%)	103 (19.4%)	< 0.001
55–59	2593 (20.1%)	2083 (20.3%)	442 (18.4%)	68 (22.9%)		4950 (20.0%)	4050 (20.3%)	787 (18.1%)	113 (21.3%)	
60–64	2833 (21.8%)	2249 (21.9%)	520 (21.6%)	64 (21.6%)		5325 (21.4%)	4318 (21.7%)	895 (20.6%)	112 (21.1%)	
65–69	2845 (21.9%)	2254 (22.0%)	543 (22.6%)	48 (16.2%)		5176 (21.9%)	4161 (20.9%)	925 (21.3%)	90 (17.0%)	
70–74 (75)^a^	2416 (18.6%)	1817 (17.7%)	550 (22.9%)	49 (16.5%)		4926 (19.9%)	3711 (18.6%)	1103 (25.4%)	112 (21.1%)	
**Sex**										
Female	12,965 (100%)	10,261 (100%)	2407 (100%)	297 (100%)	–	13,345 (53.9%)	10,543 (52.9%)	2499 (57.6%)	303 (57.2%)	< 0.001
Male	–	–	–	–		11,437 (46.1%)	9371 (47.1%)	1839 (42.4%)	277 (42.8%)	
**Race-ethnicity**^**b**^										
NHW	9413 (72.6%)	7453 (72.6%)	1744 (72.5%)	216 (72.7%)	= 0.457	18,241 (73.6%)	14,667 (73.6%)	3205 (73.9%)	369 (69.6%)	= 0.005
NHB	1483 (11.4%)	1159 (11.3%)	295 (12.3%)	29 (9.8%)		2690 (10.9%)	2104 (10.6%)	519 (12.0%)	67 (12.6%)	
NHO	808 (6.3%)	656 (6.4%)	135 (5.6%)	17 (5.7%)		1562 (6.3%)	1281 (6.4%)	248 (5.8%)	33 (6.2%)	
Hispanic	1261 (9.7%)	993 (9.7%)	233 (9.7%)	35 (11.8%)		2289 (9.2%)	1862 (9.4%)	366 (8.4%)	61 (11.5%)	
**Education**^**c**^										
≤ HS	4482 (34.7%)	3362 (32.9%)	980 (40.9%)	140 (47.5%)	< 0.001	8863 (35.9%)	6748 (34.0%)	1842 (42.7%)	273 (52.0%)	< 0.001
> HS	8436 (65.3%)	6866 (67.1%)	1415 (59.1%)	155 (52.5%)		15,804 (64.1%)	13,083 (66.0%)	2469 (57.3%)	252 (48.0%)	
**Marital status**										
Single	1203 (9.4%)	928 (9.2%)	238 (10.0%)	37 (12.6%)	< 0.001	2593 (10.6%)	2002 (10.2%)	505 (11.8%)	86 (16.5%)	< 0.001
Live with spouse	6628 (51.9%)	5479 (54.2%)	1049 (44.1%)	100 (34.0%)		13,617 (55.8%)	11,384 (58.0%)	2031 (47.6%)	202 (38.8%)	
Other^d^	4945 (38.7%)	3699 (36.6%)	1089 (45.8%)	157 (53.4%)		8195 (33.6%)	6229 (31.8%)	1733 (40.6%)	233 (44.7%)	
**Insurance coverage**										
Insured	12,221 (94.5%)	9668 (94.4%)	2278 (94.9%)	275 (92.6%)	= 0.249	23,251 (94.0%)	18,643 (93.8%)	4109 (94.9%)	499 (94.3%)	= 0.033
Uninsured	714 (5.5%)	569 (5.6%)	123 (5.1%)	22 (7.4%)		1475 (6.0%)	1223 (6.2%)	222 (5.1%)	30 (5.7%)	
**Cancer diagnosed**										
Yes	2205 (17.0%)	1591 (15.5%)	553 (23.0%)	61 (20.5%)	< 0.001	3981 (16.1%)	2952 (14.8%)	926 (21.4%)	103 (19.4%)	< 0.001
No	10,747 (83.0%)	8658 (84.5%)	1853 (77.0%)	236 (79.5%)		20,772 (83.9%)	16,935 (85.2%)	3410 (78.6%)	427 (80.6%)	
**Chronic disease score**^**e**^	1.85 ± 1.60	1.62 ± 1.48	2.62 ± 1.75	3.12 ± 1.96	< 0.001	1.90 ± 1.63	1.70 ± 1.51	2.67 ± 1.81	3.22 ± 1.02	< 0.001

^a^Mammogram 50–74; Colonoscopy 50–75

^*b*^*NHW* Non-Hispanic White, *NHB* Non-Hispanic Black, *NHO* Non-Hispanic Other

^c^ < = HS: High school or less; > HS: More than high school

^d^Widowed/Divorced/Separated/Never married

^e^Mean ± Std; Included chronic disease: Hypertension, High cholesterol, Coronary heart disease, Angina, Myocardial infarction, Stroke, Chronic obstructive pulmonary disease, Asthma, Diabetes, Arthritis

^f^Based on weighted chi-square test or one-way Anova test

Among the 24,782 individuals eligible for colonoscopy, 4868 (or 19.6%) were cognitively impaired, with 4338 (17.5%) and 530 (2.1%) having mild and severe cognitive impairment, respectively. Overall, 53.9% of the individuals were female, 73.6% were Non-Hispanic White, 64.1% had more than high school education, 55.8% were living with spouse, 94.0% had health insurance, and 16.1% had a prior cancer diagnosis. We observed a significant difference in each of the characteristic by cognitive impairment status. Compared to the cognitively unimpaired individuals, those with impairment were more likely to be female (57.6% or 57.2% in the mildly or severely impaired vs. 52.9% in the unimpaired; *p* <  0.001), Non-Hispanic Black (12.0% or 12.6% vs. 10.6%; *p* = 0.005), have high school or less education (42.7% or 52.0% vs. 34.0%; *p* <  0.001), be single (11.8% or 16.5% vs. 10.2%; *p* <  0.001), be insured (94.9% or 94.3% vs. 93.8%; *p* = 0.033), have a prior cancer diagnosis (21.4% or 19.4% vs. 14.8%; *p* <  0.001), and have higher number of chronic diseases (2.67 or 3.22 vs. 1.70; *p* <  0.001).

### Cancer screening utilization by cognitive impairment status

We summarized the percentage of mammogram and colonoscopy use in our study populations stratified by cognitive impairment status in Table 2. As shown in the table, the overall percentage of mammogram use in women aged 50–74 years was 72.9% (95% confidence interval [CI] = 71.4–74.3%) and 76.5% (95% CI = 75.3–77.7%) in 2018 and 2019, respectively. The percentage of mammogram use in cognitively impaired women was significantly lower than that in women without cognitive impairment in both 2018 (73.7% in the unimpaired vs. 71.6% or 58.3% in the mildly or severely impaired; *p* = 0.002) and 2019 (77.2% vs. 74.1% or 66.1%; *p* = 0.008). The overall percentage of colonoscopy use in adults aged 50–75 years was 60.6% (95% CI: 59.4–61.9%) and 57.9% (95% CI: 56.8–58.9%) in 2018 and 2019, respectively. The percentage of colonoscopy use in cognitively impaired individuals was slightly higher than that in the cognitively unimpaired individuals in both 2018 (60.3% vs. 61.8% or 63.6%; *p* = 0.436) and 2019 (57.8% vs. 58.3% or 56.9%; *p* = 0.918), although the differences were statistically non-significant.

**Table 2:** Percentages of cancer screening use (95% confidence interval) by cognitive impairment status among screening-eligible populations

Cancer screening		Cognitive impairment			***p***-value
2018					
	**Overall (*n*** **= 5687)**	**Severely impaired** **(*n*** **= 157)**	**Mildly impaired (*n*** **= 1102)**	**Unimpaired (*n*** **= 4428)**	
**Mammogram**	72.9% (71.4–74.3%)	58.3% (48.0–68.6%)	71.6% (68.3–74.8%)	73.7% (72.1–75.4%)	= 0.002
	**Overall (n = 10,811)**	**Severely impaired** **(*n*** **= 259)**	**Mildly impaired (*n*** **= 1980)**	**Unimpaired (*n*** **= 8572)**	
**Colonoscopy**	60.6% (59.4–61.9%)	63.6% (56.2–70.9%)	61.8% (59.0–64.6%)	60.3% (59.0–61.6%)	= 0.436
2019					
	**Overall (*n*** **= 7278)**	**Severely impaired** **(*n*** **= 140)**	**Mildly impaired (*n*** **= 1305)**	**Unimpaired (*n*** **= 5833)**	
**Mammogram**	76.5% (75.3–77.7%)	66.1% (56.6–75.6%)	74.1% (71.2–77.4%)	77.2% (75.9–78.6%)	= 0.008
	**Overall (n = 13,971)**	**Severely impaired** **(*n*** **= 271)**	**Mildly impaired (*n*** **= 2358)**	**Unimpaired (*n*** **= 11,342)**	
**Colonoscopy**	57.9% (56.8–58.9%)	56.9% (49.3–64.5%)	58.3% (55.6–60.9%)	57.8% (56.7–58.9%)	= 0.918

### Multivariable analysis

We summarized results from the multivariable logistic model on the association of cognitive impairment with recent mammogram attendance in Table 3. Compared to the cognitively unimpaired women, the cognitively impaired women, mild (odds ratio [OR] = 0.85; 95% CI = 0.74–0.97) or severe (OR = 0.54; 95% CI = 0.38–0.76), were less likely to have had a recent mammogram adjusting for the covariates. We did not observe an overall age effect in having a mammogram. However, women aged 65–69 years were more likely to have had a recent mammogram compared to women aged 50–54 years (OR = 1.20; 95% CI = 1.02–1.41). Respondents more likely to have had a recent mammogram included those who were Non-Hispanic Black (OR = 1.38; 95% CI = 1.16–1.66) and Hispanic (OR = 1.51; 95% CI = 1.25–1.83) (reference = Non-Hispanic White), had more than high school education (OR = 1.47; 95% CI = 1.32–1.63), were living with spouse (OR = 1.51; 95% CI = 1.36–1.68), were insured (OR = 4.22; 95% CI = 3.46–5.16) and had a prior cancer diagnosis (OR = 1.18; 95% CI = 1.03–1.37).

**Table 3:** Multivariable logistic regression estimating association between cognitive impairment and mammogram utilization among 50–74 years old female in NHIS

	Female (***N*** = 12,615)	
Variables	Adjusted OR (95% CI)	***p***-value
**Cognitive impairment**		
Mild vs. No impairment	0.85 (0.74, 0.97)	= 0.015
Severe vs. No impairment	0.54 (0.38, 0.76)	< 0.001
**Age**		
55–59 vs. 50–54	1.11 (0.94, 1.30)	= 0.220
60–64 vs. 50–54	1.11 (0.94, 1.30)	= 0.209
65–69 vs. 50–54	1.20 (1.02, 1.41)	= 0.031
70–74 vs. 50–54	1.02 (0.86, 1.22)	= 0.804
**Race-ethnicity**^**a**^		
NHB vs. NHW	1.38 (1.16, 1.66)	< 0.001
NHO vs. NHW	0.84 (0.69, 1.03)	= 0.097
Hispanic vs. NHW	1.51 (1.25, 1.82)	< 0.001
**Education**^**b**^		
> HS vs < = HS	1.47 (1.32, 1.63)	< 0.001
**Marital**		
Live with spouse vs. Other^c^	1.51 (1.36, 1.68)	< 0.001
Single vs. Other	0.94 (0.78,1.12)	= 0.481
**Insurance coverage**		
Insured vs. Uninsured	4.22 (3.46, 5.16)	< 0.001
**Cancer diagnosed**		
Yes vs. No	1.18 (1.03, 1.37)	= 0.021
**Chronic disease score**^**d**^	1.07 (1.04, 1.12)	< 0.001

^a^*NHW* Non-Hispanic White, *NHB* Non-Hispanic Black, *NHO* Non-Hispanic Other

^b^ < = HS: High school or less; > HS: More than high school

^c^Widowed/Divorced/Separated/Never married

^d^Included chronic disease: Hypertension, High cholesterol, Coronary heart disease, Angina, Myocardial infarction, Stroke, Chronic obstructive pulmonary disease, Asthma, Diabetes, Arthritis

We summarized results from the multivariable logistic model on the association of cognitive impairment with having a colonoscopy in Table 4. Since we found multiple significant sex by covariate interactions, including sex by cognitive impairment interaction, we examined potential effect modification by fitting separate logistic models for men and women. Among male respondents, compared to the cognitively unimpaired men, the cognitively impaired men, mild (OR = 0.79; 95% CI = 0.69–0.91) or severe (OR = 0.69; 95% CI = 0.48–0.98), were less likely to have had a recent colonoscopy adjusting for the covariates. We observed an overall age effect in having a colonoscopy, with older men being more likely to have had a recent test. We did not observe a significant difference in having a recent colonoscopy between Non-Hispanic White and Non-Hispanic Black respondents. However, Non-Hispanic Other (OR = 0.55; 95% CI = 0.45–0.67) and Hispanic (OR = 0.62; 95% CI = 0.52–0.74) respondents were less likely to have had a recent colonoscopy compared to Non-Hispanic White respondents. In addition, respondents who had higher than high school education (OR = 1.51; 95% CI = 1.36–1.67), were living with spouse (OR = 1.55; 95% CI = 1.38–1.73), were insured (OR = 3.07; 95% CI = 2.45–3.85), had a prior cancer diagnosis (OR = 1.60; 95% CI = 1.38–1.85), and had more chronic diseases (OR = 1.15; 95% CI = 1.11–1.19) were more likely to have had a recent colonoscopy.

**Table 4:** Multivariable Logistic regression estimating association between cognitive impairment and colonoscopy utilization among 50–75 years old female and male in NHIS

	Male (N = 11,084)		Female (N = 13,002)	
Variables	Adjusted OR (95% CI)	***p***-value	Adjusted OR (95% CI)	***p***-value
**Cognitive impairment**				
Mild vs. No impairment	0.79 (0.69, 0.91)	< 0.001	1.02 (0.91, 1.15)	= 0.699
Severe vs. No impairment	0.69 (0.48, 0.98)	= 0.038	1.33 (0.97, 1.82)	= 0.080
**Age**				
55–59 vs. 50–54	2.16 (1.86, 2.52)	< 0.001	1.91 (1.66, 2.21)	< 0.001
60–64 vs. 50–54	2.30 (1.97, 2.67)	< 0.001	2.36 (2.05, 2.73)	< 0.001
65–69 vs. 50–54	2.39 (2.04, 2.80)	< 0.001	2.37 (2.06, 2.74)	< 0.001
70–74 vs. 50–54	2.45 (2.08, 2.90)	< 0.001	2.46 (2.12, 2.85)	< 0.001
**Race-ethnicity**^**a**^				
NHB vs. NHW	1.05 (0.89, 1.24)	= 0.552	1.12 (0.96, 1.31)	= 0.136
NHO vs. NHW	0.55 (0.45, 0.67)	< 0.001	0.76 (0.63, 0.91)	= 0.002
Hispanic vs. NHW	0.62 (0.52, 0.74)	< 0.001	0.81 (0.69, 0.94)	= 0.006
**Education**^**b**^				
> HS vs < = HS	1.51 (1.36, 1.67)	< 0.001	1.45 (1.32, 1.60)	< 0.001
**Marital**				
Live with spouse vs. Other^c^	1.55 (1.38, 1.73)	< 0.001	1.46 (1.32, 1.61)	< 0.001
Single vs. Other	0.83 (0.70, 0.98)	= 0.030	0.94 (0.80, 1.12)	= 0.487
**Insurance coverage**				
Insured vs. Uninsured	3.07 (2.45, 3.85)	< 0.001	3.12 (2.54, 3.85)	< 0.001
**Cancer diagnosed**				
Yes vs. No	1.60 (1.38, 1.85)	< 0.001	1.47 (1.30, 1.67)	< 0.001
**Chronic disease score**^**d**^	1.15 (1.11, 1.19)	< 0.001	1.12 (1.08, 1.15)	< 0.001

^*a*^*NHW* Non-Hispanic White, *NHB* Non-Hispanic Black, *NHO* Non-Hispanic Other

^b^ < = HS: High school or less; > HS: More than high school

^c^Widowed/Divorced/Separated/Never married

^d^Included chronic disease: Hypertension, High cholesterol, Coronary heart disease, Angina, Myocardial infarction, Stroke, Chronic obstructive pulmonary disease, Asthma, Diabetes, Arthritis

Among female respondents, compared to the cognitively unimpaired women, the cognitively impaired women, mild (OR = 1.02, 95% CI = 0.91–1.15) or severe (OR = 1.33; 95% CI = 0.97–1.82), were slightly more likely to have had a recent colonoscopy adjusting for the covariates, although the difference was statistically non-significant. We observed an overall age effect in having a colonoscopy, with older women being more likely to have had a recent test. Similar to results in the male respondents, we did not observe a significant difference in having a recent colonoscopy between Non-Hispanic White and Non-Hispanic Black respondents. However, Hispanic (OR = 0.81; 95% CI = 0.69–0.94) and Non-Hispanic Other (OR = 0.76; 95% CI = 0.63–0.91) respondents were less likely to have had a recent colonoscopy compared to their Non-Hispanic White counterparts. In addition, respondents who had more than high school education (OR = 1.45; 95% CI = 1.32–1.60), were living with spouse (OR = 1.46; 95% CI = 1.32–1.61), were insured (OR = 3.12; 95% CI = 2.53–3.84) had a prior cancer diagnosis (OR = 1.47; 95% CI = 1.30–1.67) and had more chronic diseases (OR = 1.12; 95% CI = 1.08–1.15) were more likely to have undergone recent colonoscopy.

## Discussion

### Principal findings

In this study, using the NHIS data, we calculated and compared the percentage of mammogram and colonoscopy use in cognitively impaired (mild or severe) versus unimpaired individuals. We found that the percentage of mammogram use was lower in cognitively impaired women compared to cognitively unimpaired women. Based on Table 2, we did not observe a significant difference in the percentage of colonoscopy use in cognitively impaired versus unimpaired individuals. In the multivariable analyses, being cognitively impaired (mild or severe) was associated with lower likelihood of having a recent mammogram in screening eligible women. Being cognitively impaired (mild or severe) was associated with lower likelihood of having a recent colonoscopy in screening eligible men, but the association was non-significant in screening eligible women.

### Cancer screening in the cognitively impaired population

A few prior studies have examined the percentage of mammogram or colonoscopy use in cognitively impaired individuals using historical NHIS data [24–26]. The earliest percentage of mammogram use was examined by Legg et al. [24], who reported a percentage of 44.0% in cognitively impaired women in 1998. But the study was restricted to women aged 50 years or older who received a mammogram within 1 year. Using a similar study population (i.e., women aged 50–74 years with a mammogram within 2 years), Iezzoni et al. examined the percentage of mammogram use in screen eligible women without breast cancer history and reported that the percentage was 63.4, 75.1, 67.6, 62.7, 65.9, and 63.0% in cognitively impaired women in 1998–2010, respectively [25]. In the most recent study, Steele et al. reported a recent mammogram use percentage of 61.2%, the lowest in 20 years, in screen eligible but cognitively impaired women 2013 [26]. However, cognitive impairment was not associated with having a mammogram in the multivariable analysis in Steele et al. Our mammogram use percentage based on 2018 and 2019 NHIS data were higher than the historical percentages, suggesting a potential upward trend of mammogram utilization in cognitive impaired women. Furthermore, unlike the Steele et al. study, the association between cognitive impairment and having a mammogram was significant in our study, independent of the covariates. For colonoscopy, Steele et al. reported that the percentage of “up-to-date” colorectal cancer screening was 56.2% in screening eligible cognitively impaired individuals, among whom 93.6% reported having had a colonoscopy. However, Steele et al. did not examine any sex differences and found that cognitive impairment was not associated with being “up-to-date” with colorectal cancer screening in the multivariable analysis. It is possible that achieving compliance with colonoscopy (with an associated 10-year window after the procedure to be up-to-date) rather than the requirement for biennial mammograms is easier for individuals with cognitive impairment.

The percentage of mammogram use has also been reported in the cognitively impaired population using data sources other than the NHIS [13, 27–29], although we did not find any report on the percentage of colonoscopy use in this population. Two studies analyzed the utilization of mammogram using Medicare claims data. Ives et al. reported a mammogram use percentage of 32.0% in cognitively impaired women aged 65–79 residing in 5 rural Pennsylvania counties. However, the percentage was based on data from almost 30 years ago in 1991–1992 [27]. Mehta et al. examined the percentage of mammogram use in cognitively impaired women aged 70 years or older using 2002 Health and Retirement Study data. The percentage was reported to be 33% among mildly-to-moderately impaired women and 18% among severely impaired women. Mehta et al. found that the severely impaired women were less likely to have had mammogram compared to cognitively unimpaired women [13]. Two other studies analyzed the utilization of mammogram using survey data. Persky & Burack conducted a telephone survey in 1989 and reported a mammogram percentage of 29% in cognitively abnormal women aged 55 or older [28]. Ostbye et al. analyzed the Asset and Health Dynamics Among the Oldest Old (AHEAD) survey data and found the percentage of mammogram use in the past 2 years was 40 and 48% in cognitively impaired women aged 70 years or more in 1995 and 2000, respectively. Ostbye et al. also found that cognitive impairment was associated with a lower likelihood of receiving a mammogram [29]. Unlike our study, most of these reports focused on the elderly population aged 65 or older and did not use nationally representative data.

### Study limitations and strengths

Our study has several limitations. First, both cognitive impairment and cancer screening use are self-reported, resulting in potential misclassification, volunteer and recall bias. Questions related to concentration and memory recall were used to define cognitive impairment which could introduce other non-ADRD etiologies that could impact cancer screening compliance rates (e.g., alcoholism). Prior studies showed that self-reports may overestimate mammogram and colonoscopy screening utilization [30, 31], and other studies found an inaccuracy of self-reported cognitive impairment [32, 33]. Additionally, the response rate of 53.1 and 59.1% for the NHIS Sample Adult component may have posed a selection bias. Finally, potential factors associated with cancer screening utilization may not have been fully controlled due to the limitation of dataset. Information on other factors, such as physician access barriers, cost/pain/safety concern and other comorbid conditions, were not recorded in the NHIS and therefore were not analyzed. Despite the limitations, this study provides data to support plausible hypotheses that are capable of being answered through additional analyses and more clinical research. Given the overlapping intersection of large and growing populations of patients with both cancer and cognitive impairment, this line of inquiry is important for establishing benchmarking data before interventions aimed to improve patient outcomes are deployed.

## Conclusion

Our study found a lower likelihood of receiving mammogram and colonoscopy in the screening eligible, cognitively impaired population. More studies are needed to examine the contributory factors associated with the difference in cancer screening utilization in this vulnerable population. Our results highlight the need for more future research on the utilization and effectiveness of cancer screening in individuals with cognitive impairment.

## Data Availability

The datasets generated and analysed during the current study are available in the National Center for Health Statistics repository, https://www.cdc.gov/nchs/nhis/data-questionnaires-documentation.htm
